# Individual and combined effect of *TP53, MDM2, MDM4, MTHFR, CCR5,* and *CASP8* gene polymorphisms in lung cancer

**DOI:** 10.18632/oncotarget.22756

**Published:** 2017-11-29

**Authors:** Ausra Stumbryte, Zivile Gudleviciene, Gabrielis Kundrotas, Daiva Dabkeviciene, Agne Kunickaite, Saulius Cicenas

**Affiliations:** ^1^ Biobank, National Cancer Institute, LT-08660 Vilnius, Lithuania; ^2^ Institute of Biosciences, Vilnius University Life Sciences Center, LT-10257 Vilnius, Lithuania; ^3^ Department of Human and Medical Genetics, Faculty of Medicine, Vilnius University, LT-08661 Vilnius, Lithuania; ^4^ Department of Thoracic Surgery and Oncology, National Cancer Institute, LT-08660 Vilnius, Lithuania; ^5^ Faculty of Medicine, Vilnius University, LT-03101 Vilnius, Lithuania

**Keywords:** lung cancer prognostic markers, SNPs, patient survival, mortality rate, HPV phylogenetic line

## Abstract

Lung cancer (LC) is the second common and with the highest mortality oncological disease. Specific biomarkers for its diagnostics, treatment, and prognosis are still under the investigations. Aim of our study was to evaluate the relationship between the polymorphisms of TP53 pathway genes *TP53*, *MDM2*, *MDM4*, the polymorphisms of HPV-associated genes *MTHFR*, *CASP8*, *CCR5*, and HPV infection with survival of LC patients. SNPs were genotyped using PCR-RFLP. qRT-PCR was used to detect, identify, and quantify HPV. No statistically significant differences were detected between individual SNPs and patient survival with stage I-IV LC. Cluster analysis of SNPs in genes *MDM4* A/A, *CCR5* wt/Δ32, *MTHFR* C/T, *MDM2* T/T showed possible association with the worse survival. Patients who were diagnosed with C/T polymorphic variant of gene *MTHFR* tend not to survive stage III-IV LC (*P =* .12). There is a tendency between MDM2 gene T/T variant and worse survival of patients diagnosed with late stage LC (*P =* .11). HPV infection is very rear among LC patients (3 of 92). Overall, there is a link, although statistically insignificant, between specific SNPs and LC patient survival frequency and time, meanwhile the combination of specific SNPs showed a statistically significant measure. In conclusion, we determined statistically significant (*P =* .04) link between the poor survival of LC patients after surgery and the combination of polymorphic variants C/T of the *MTHFR* and T/T of the *MDM2* genes, whereas individually these SNPs do not show significant relationship with the survival of patients after surgery.

## INTRODUCTION

Lung cancer (LC) is one of the most common malignant tumors [1] and the leading cause of all cancer related deaths worldwide [2]. It is characterized by uncontrolled growth of cells comprising pulmonary tissue, which causes a significant decline in human health and life expectancy [1]. According to the World Health Organization, about 1.8 million people (1.240.600 men and 583.100 women) all over the world were newly diagnosed with LC in 2012, which represents 12.7% of all new cancer cases that year. During the same year LC resulted in death at least 1.59 million times (1.098.700 male and 491.200 female) globally, which is 18.2% of all deaths caused by cancer per annum [2, 3]. Epidemiological data on LC in Lithuania reflects similar tendencies with 1421 new cases (8% of all cancers) and 1355 deaths (16.97%) in 2012 [4].

There are two main pathological types of LC: small-cell lung cancer (SCLC) and non-small-cell lung cancer (NSCLC). NSCLC is the most common form of LC, where about 85% of cancers are classified as NSCLC, while SCLC occurs in approximately 13–15% of patients [2]. The main symptoms of this disease are coughing, fatigue, dyspnea, and hemoptysis. These symptoms hinder diagnosis, since patients usually have a confused idea of the causes, therefore the early stages of the disease (stages I and II) are hardly ever detected [2, 5]. Due to these reasons, most of LC patients usually are diagnosed with an advanced stage (III–IV) and non-resectable disease [6]. About 40% of patients are diagnosed with LC in stage IV of the disease, and approximately 30% in stage III. The fact that the disease is diagnosed in its advanced stages reduces the patient’s survival rate (5 years) to 16% [2, 7].

It is well known that tobacco is considered to be the main risk factor for LC development. The length of time spent smoking and type of tobacco used may increase the relative risk of acquiring this disease [2, 8, 9]. Although smoking is the major etiologic factor, not all smokers develop LC. Moreover, approximately 15–25% of those with LC have never been smokers. Studies of genes candidates and genome-wide association studies have revealed many dysfunctional genes that might be associated with this type of cancer indicating that LC is caused by both genetic and environmental factors, and their interactions as well [1, 10, 11]. Although exposure to carcinogens is considered to be the main cause, there are the new data on Human Papillomavirus (HPV) infection and LC: individual genetic variation and interaction with HPV may contribute to lung cancer development [12].

**Figure 1:**
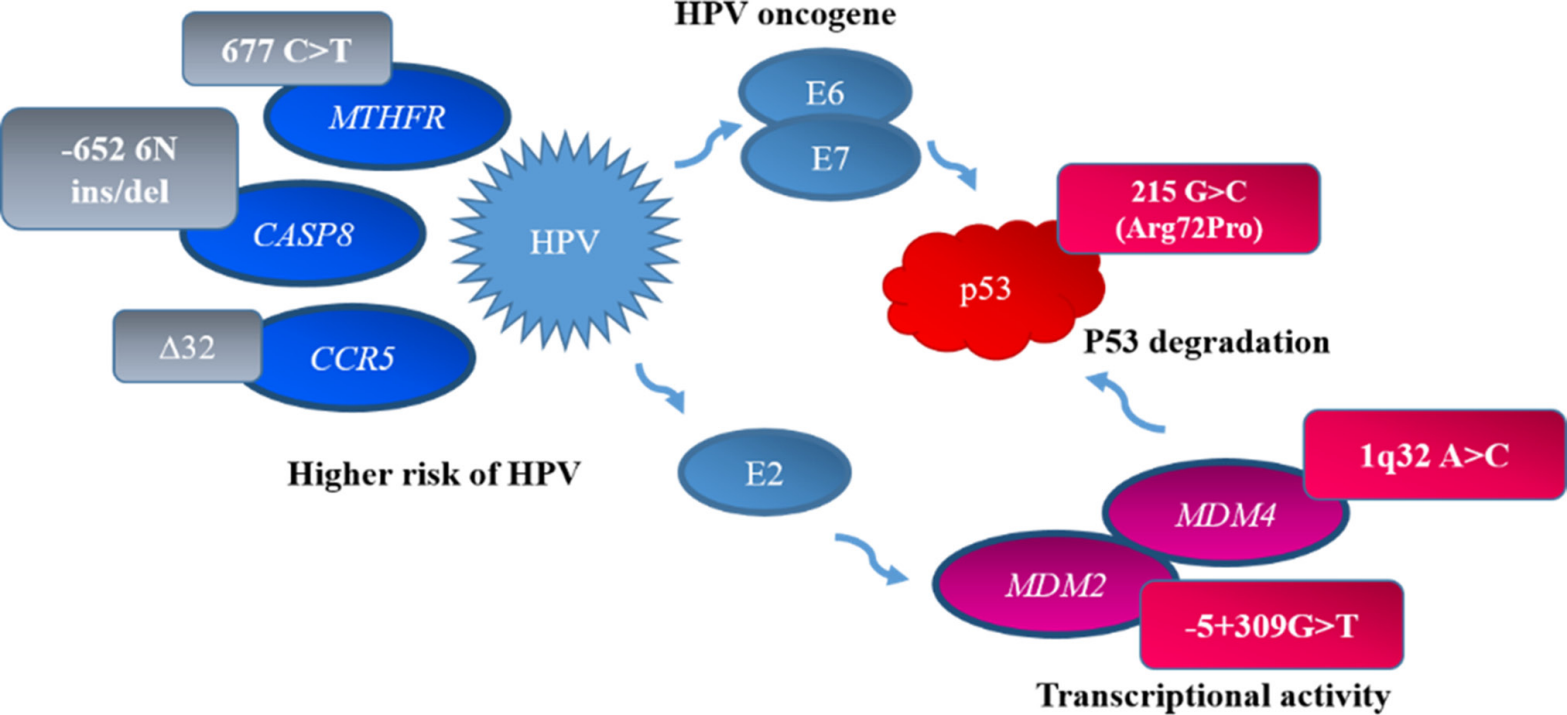
Gene and HPV interactions p53 is inactivated by HPV E6 protein. This action disturbs cell cycle that generates opportunities for new mutations. HPV E2 protein interacts with *MDM2/MDM4* encoded proteins that are concentrated near HPV16 promoter, where these proteins encourage E2 managed transcription activity. Polymorphic variants of genes *MTHFR* 677 C > T, *CASP8*-652 6N ins/del, and *CCR5* Δ32 are associated with increased susceptibility to HPV infection.

However, not only tobacco smoking or various infections are responsible for LC development. Various genes and their mutations are closely associated with LC. Large studies were performed to find the exact biomarkers for precise diagnostic and treatment or follow-up of LC patients. On the other hand, multiple biologically relevant polymorphisms may have more accurate prediction power of cancer prognosis compared with single polymorphism because of the modest effect [13]. The functional polymorphisms in genes *TP53* (rs1042522), *MDM2* (rs2279744), *MDM4* (rs4245739), *MTHFR* (rs1801133), *CASP8* (rs3834129), and *CCR5* (rs333) alone or in combination, could affect survival in advanced non-small cell lung cancer (NSCLC) patients [13] (Figure 1).

Combined effect of HPV infection and gene polymorphism could have an important role in the development of NSCLC. Recently, it has been reported that high-risk HPV 16/18 3E6 protein is associated with p53 protein degradation in LC [14]. The tumor suppressor *TP53* pathway plays a crucial role in preventing carcinogenesis through its ability to impose cell cycle arrest and apoptosis following DNA damage and oncogene activation [15, 16]. The HPV oncogenic protein E6 has a strong binding affinity for p53 leading to its ubiquitination and degradation, resulting in reduced protein function and loss of cell cycle control [17]. Multiple functional single nucleotide polymorphysms (SNPs) occur in the *TP53* gene and the most frequently studied is the G to C change at codon 72 in exon 4, resulting in the amino acid substitution of Arg to Pro (Arg72Pro, rs1042522). This SNP has been shown to alter *TP53* function because it is located in the proline-rich region that is required for *TP53* to induce apoptosis. Emerging evidence has shown that the *TP53* Arg/Pro polymorphism is not only associated with LC development, but also affects individual sensitivity to platinum-drug chemotherapy and patients’ survival [13]. A tendency for a worse prognosis is seen in patients with the Pro/Pro and Arg/Arg genotypes than in those with the Arg/Pro genotype [3, 5]. Recently, *in vitro* and *in vivo* studies have indicated a greater susceptibility to degradation by HPV E6 for the p53 protein produced by the Arg allele at codon 72 than for the p53 protein produced by the Pro allele [7].

In most, if not all, cancers lacking mutation, wild-type p53 is inactivated by interaction with cellular (*MDM2*/*MDM4*) or viral proteins, leading to its degradation [18]. The murine double minute 2 gene *(MDM2*) is a key negative regulator of the *TP53* pathway and usually is overexpressed in many cancers as oncoprotein. MDM2 oncogenic protein is the principal cellular antagonist of the *TP53* gene product [19]. DNA damage signals phosphorylation of MDM2 to cause protein structure changes that stabilize p53 resulting in progression through the cell cycle. Moreover, mechanistically MDM2 interacts with HPV E2 protein to synergistically activate the HPV16 promoter, demonstrating E2 can actively recruit MDM2 to the HPV promoter and supporting a role for MDM2 in the transcriptional activity of HPV [17]. A *MDM2* SNP at the 309th nucleotide in the first intron (rs2279744), with a T to G change, could increase the affinity for stimulatory protein 1 binding and result in increased *MDM2* expression and subsequent attenuation of the *TP53* pathway. This polymorphism has been associated with several cancers including LC [20].

The MDM4 protein plays an important role in the negative regulation of the TP53 through its interaction with MDM2. *MDM4* amplification has been observed in several tumor forms. A polymorphism (rs4245739 A>C; SNP34091) in the *MDM4* 3′ untranslated region has been reported to create a target site for *hsa-miR-191*, resulting in decreased *MDM4* mRNA levels [21]. Therefore, *MDM4*, together with *TP53*, *MDM2* and HPV E6 oncoprotein, may play a critical role in HPV-associated carcinogenesis [17, 22] and lung cancer as well.

Folate is essential for the synthesis of nucleotides, its deficiency induces double-strand breaks and increases cancer risk [23]. Methylene tetrahydrofolate reductase (encoded by *MTHFR*) is a key enzyme in metabolism of folate and nucleotides to maintain DNA stability and prevent cancers through catalysing irreversible conversion of 5,10-methylenetetrahydrofolate (5, 10-methyleneTHF) to 5-methyl tetrahydrofolate (5-methyl THF) [24, 25, 26]. The *MTHFR* gene is located on the short arm of chromosome 1 (1p36.3) and its cDNA has a total length of 2.2 kb [27]. *MTHFR* is involved in DNA methylation and synthesis as an important enzyme in the folic acid metabolic process [28]. The most common mutation is a C to T transition at nucleotide 677 (rs1801133, C677T) in exon 4, resulting in a substitution of alanine with valine [29]. The *MTHFR* T/T genotype exhibited a significantly increased risk of LC compared to the *MTHFR* C/C and C/T genotype [28].

Caspase-8 (encoded by the *CASP8* gene) is crucial in generating cell death signals and eliminating potentially malignant cells. Genetic variation in *CASP8* may affect susceptibility to cancer [30, 31]. Caspase-8 is a member of the caspase cysteine protease family and plays an important role in cancer development [32]. *CASP* causes cell death by nuclear membrane breakdown, DNA fragmentation, chromatin condensation, and the formation of apoptotic genetic polymorphisms for genes controlling the cell cycle or apoptosis [1, 33]. Recently, a large number of SNPs in the *CASP* apoptotic pathway have been increasingly recognized. Several studies have demonstrated that some variants in apoptosis pathway genes are associated with the susceptibility to various human cancers, especially LC [1, 34]. The *CASP8*-652 6N ins/del (rs3834129) polymorphism has been previously reported to influence the progression to several cancers. Finally, this suggests that the del allele, carrier and ins/del genotype of the -652 6N ins/del polymorphism in the *CASP8* gene may be protective factors for cancer development [30].

Chemokines are a family of small cytokines that regulate leukocytes in tissues, thus playing an important role in regulation of immunological processes [35]. Chemokines participate in many pathophysiological conditions, including malignant tumors progression and metastasis. Role of these substances in malignancies are complex. In addition to their chemotactic properties, chemokines and their receptors also play a part in other biological functions relevant to oncogenesis or they indirectly affect tumor development by attracting immunocompetent cells with pro- or anti-tumoral activities [36]. The *CCR5*-Δ32 polymorphism (rs333), which is a 32-bp deletion in the coding region, encodes a truncated inactive receptor that is not expressed on the cell membrane. Moreover, molecular epidemiological studies showed that Δ32 polymorphisms are associated with cancer development [37].

HPVs are small non-enveloped DNA viruses that usually infect squamous epithelial cells [38]. It is well known that HPV is a major risk factor for cervical, anal, vaginal, skin or laryngeal cancers. According to the previous investigations, HPV infection is local and develops only in epithelium. However, there are new data on findings of HPV in breast, prostate, and lung cancers [39, 40]. These findings paved the way to the idea on HPV transmission through blood or lymph. Another hypothesis states that the virus enters through an oral cavity and spreads among its cells to the lung [2]. While investigating the presence of HPV in the exhaled breath condensate of LC patients [2, 40], high risk types of HPV were found. These findings also suggest a possibility of air stream transmission of HPV. For a long time, it was believed that papillomavirus could not migrate to the other parts of the body through the blood because HPV has not a viremic phase during active infection [2]. However, several studies have reported LC-related HPV infection rates that vary between 10% and 80%, depending on the different methods used or specific geographical regions investigated [38]. After analysis gathered from global research reports, it was found that 22.4% of the patients with LC could be presented with an HPV infection, suggesting that HPV infection could be one of the important risk factors to the tumorigenesis of NSCLC [38, 41].

Summing up, all these above described genes or SNPs alone or in combination could affect survival of patients with advanced LC. In this study, we for the first time investigated the impact of a set of HPV and such different SNPs as *TP53* Arg72Pro (rs1042522), *MDM2* T309G (rs2279744), *MDM4* (rs4245739), *MTHFR* (rs1801133), *CASP8* (rs3834129), and *CCR5* (rs333) on the advanced LC patients` survival.

## RESULTS

### Patient characteristics

The mean age of the patients included in the study was 63 years (SD 8.7). Tumor cases from 76 (82.6%) male and 16 (17.4%) female patients were collected during the surgical operation. Histological type of cancer distribute as follows: 41 squamous-cell carcinoma (45.1%), 31 adenocarcinoma (34.1%), 6 pleomorphic-cell carcinoma (6.6%), 5 large-cell carcinoma (5.5%), 4 small-cell carcinoma (4.4%), 2 carcinoid (2.2%), 1 giant-cell carcinoma (1.1%), 1 chorio-carcinoma (1.1%), one case histology type has not been established. 92 lung cancer patients were divided into groups according to the stage of diagnosis: stage I – 36 (39.1%), stage II – 28 (30.4%), stage III – 23 (25.0%), and stage IV – 5 (5.4%). Regarding the smoking status, 16 patients were non-smokers, 75 patients smoked, while in 1 case the data was not given.

### Allele and genotype distribution

In our study we investigated the *TP53* Arg72Pro (rs1042522), *MDM2* T309G (rs2279744), *MDM4* (rs4245739), *MTHFR* (rs1801133), *CASP8* (rs3834129), and *CCR5* (rs333) polymorphisms in 92 Lithuanian LC patients. After analysis the *TP53* gene Arg72Pro genotype distributions were as follows: the majority of sampled patients were identified as Arg/Pro polymorphic variant (95.7%) and only in a few samples genotype of Arg/Arg (4.4%) was found. Pro/Pro polymorphic variant was not detected in our investigated cases. The allele frequencies for *MDM2* gene (-410T-G) SNP (T/T, T/G, and G/G) were 50.0%, 43.5%, and 6.5%, respectively. *MDM4* gene A>C SNP distributions (A/A, A/C, and C/C) were recorded to be 59.8%, 32.6%, and 7.6%, respectively. *MTHFR* gene C677T SNP distribution was as follows: C/C 58.7%, C/T 36.9%, T/T 4.4%. *CASP8* gene (-652 6N ins/del) ins/ins, ins/del, and del/del genotype distribution was 28.3%, 66.3%, 5.4%, respectively. *CCR5* gene-delta32 deletion SNP distribution was: wt/wt 83.7%, wt/∆32 14.1%, ∆32/∆32 2.2%. The frequency distributions of all genotypes among patients are presented in Table 1.

**Table 1:** Genotype and stage frequencies of *TP53*, *MDM2*, *MDM4*, *MTHFR*, *CASP8*, *CCR5* in lung cancer patients

		Sex		*P*-value	Stage		*P*-value
Genotype	No (%)	Male (*n =* 76)	Female (*n =* 16)		I–II	III–IV	
***TP53*c.215 G>C** **(Arg72Pro)** **Arg/Arg** **Arg/Pro** **Pro/Pro**	4 (4.3%) 88 (95.7%) 0 (0%)	3 (3.9%) 73 (96.1%) 0 (0%)	1 (6.3%) 15 (93.7%) 0 (0%)	.54	2 (2.2%) 61 (67.1%) 0 (0%)	2 (2.2%) 26 (28.6%) 0 (0%)	.46
***MDM2*c.-5 + 309 G>T** **T/T** **T/G** **G/G**	46 (50.0%) 40 (43.5%) 6 (6.5%)	40 (52.6%) 33 (43.4%) 3 (4.0%)	6 (37.5%) 7 (43.7%) 3 (18.8%)	.08	36 (39.6%) 24 (26.4%) 3 (3.3%)	10 (11.0%) 15 (16.5%) 3 (3.3%)	.24
***MDM4*c.1q32 A>C** **A/A** **A/C** **C/C**	55 (59.8%) 30 (32.6%) 7 (7.6%)	45 (59.2%) 24 (31.6%) 7 (9.2%)	10 (62.5%) 6 (37.5%) 0 (0%)	.44	37 (40.7%) 21 (23.0%) 5 (5.5%)	17 (18.7%) 9 (9.9%) 2 (2.2%)	.45
***MTHFRc*.677 C>T** **C/C** **C/T** **T/T**	54 (58.7%) 34 (36.9%) 4 (4.4%)	42 (55.3%) 31 (40.8%) 3 (3.9%)	12 (75.0%) 3 (18.7%) 1 (6.3%)	.25	33 (36.3%) 27 (29.7%) 3 (3.3%)	20 (22.0%) 7 (7.7%) 1 (1.1%)	.18
***CASP8*c.-652 6N ins/del** **Ins/ins** **Ins/del** **Del/del**	26 (28.3%) 61 (66.3%) 5 (5.4%)	22 (28.9%) 49 (64.5%) 5 (6.6%)	4 (25.0%) 12 (75.0%) 0 (0%)	.51	15 (16.5%) 44 (48.3%) 4 (4.4%)	11 (12.1%) 16 (17.6%) 1 (1.1%)	.05
***CCR5*-∆32** **wt/wt** **wt/∆32** **∆32/∆32**	77 (83.7%) 13 (14.1%) 2 (2.2%)	64 (84.2%) 10 (13.2%) 2 (2.6%)	13 (81.3%) 3 (18.7%) 0 (0%)	.69	55 (50.5%) 7 (7,7%) 1 (1.1%)	21 (23.1%) 6 (6.6%) 1 (1.1%)	.74
	**No = 92**				No = 91^*^		

^*^For 1 patient the stage has not been evaluated, so this patient is not included in the analysis.

Subgroup analysis revealed marked relation between *MDM2* SNP distribution and patient sex (χ2 = 5.1, *P* = .08, df = 2) (Table 1). *MDM2*c.-5+309 G>T G/G polymorphic variant was detected in higher frequency (18.8%) among female patients (3 of 16 patients), while in samples of male patients accounted only for 4.0% (3 of 76 patients). T/T polymorphic variant was detected in 52.6% of male samples (40 of 70 patients) and in 37.5% of female samples (6 of 16 patients). *MTHFR*c.677 C>T C/C polymorphic variant was characterized by the greater frequency (75.0%) in women samples (12 patients out of 16), in comparison to 55.3% of male samples (42 patients out of 76). C/T polymorphic variant was detected more frequently (40.8%) in male patients (31 patients out of 76), while in female patients it amounted for 18.7% (3 of 16 female patients). However, all these differences were not statistically significant (χ^2^ = 2.8, *P* = .25, df = 2).Survival analysis according to TP53c.215 G>C (Arg72Pro), MDM2c.-5+309 G>T, MDM4c.1q32 A>C, MTHFRc.677 C>T, CASP8c.-652 6N ins/del, CCR5c.-∆32 genes SNPs

The main task of our study was to determine the relationship between polymorphic variants of genes *TP53*c.215 G>C (Arg72Pro)*, MDM2*c.-5+309 G>T*, MDM4*c.1q32 A>C*, MTHFR*c.677 C>T*, CASP8*c.-652 6N ins/del*, CCR5*-∆32 and LC patients’ survival. Due to the lack of uncensored values of Arg/Arg and Pro/Pro in SNP case, survival rate curves according to *TP53* gene Arg/Pro, Arg/Arg, and Pro/Pro SPNs were not presented. However, it should be noted that 3 (75.0%) LC patients out of 4 diagnosed with Arg/Arg polymophic variant survived until the last day of this study. Meanwhile 51 (57.9%) patients out of 88 survived until the last day of trial with Arg/Pro polymorphic variant detected.

**Figure 2:**
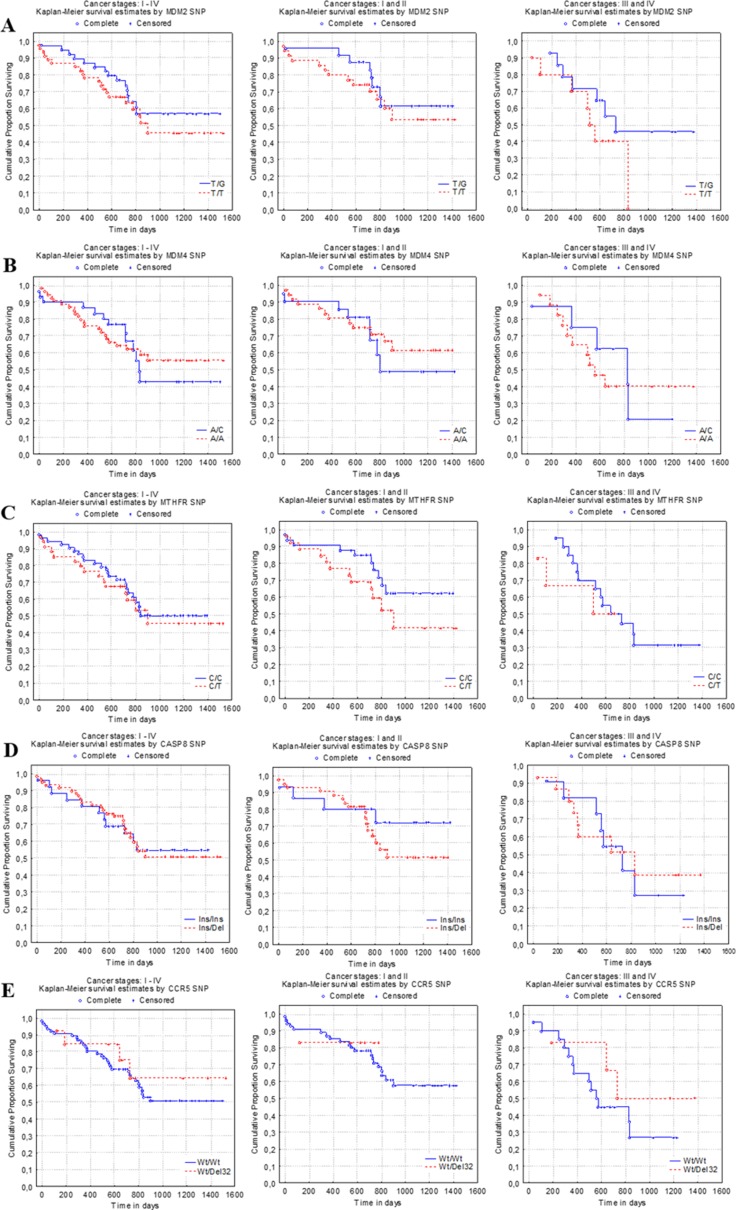
Lung cancer patient survival curves according to *MDM2, MDM4, MTHFR, CASP8*, and *CCR5* genes SNPs Patients› survival rates: (**A)** (left) - *MDM2*c.-5 + 309 G>T gene with T/T and T/G polymorphisms (*P* = .383); (**B**) (left) - *MDM4*c.1q32A>C gene with A/C, A/A polymorphisms (*P* = .836); (**C)** (left) - *MTHFR*c.677 C>T gene polymorphic C/T, C/C variants (*P* = .660); (**D)** (left) - *CASP8*c.-652 6N ins/del gene polymorphic ins/ins, ins/del variants (*P* = .986); (**E)** (left) - *CCR5*-Δ32 gene wt/Δ32, wt/wt polymorphic variants (*P* = .593). Survival curves of lung cancer patients, who were identified with: A - *MDM2*c.-5 + 309 G>T gene T/G or T/T polymorphic variants at early (centre, *P* = .489) and late (right, *P* = .227) stages; B - *MDM4*c.1q32A>C gene A/C or A/A polymorphic variant at early (centre, *P* = .606) and late (right, *P* = .866); C - *MTHFR*c.677C>T gene C/C, C/T polymorphic variants at early (centre, *P*=.165) and late (right, *P*=.728); D - *CASP8*c.-652 6N gene ins/del, ins/ins variants at early (centre, *P* = .334) and late (right, *P* = .793); E - *CCR5*-Δ32 gene wt/wt, wt/Δ32 variants at early (centre, *P* = .760) and late (right, *P* = .330) stages.

After identifying polymorphic variants of *MDM2*c.-5+309 G>T gene in the test group it was found that patients with T/G polymorphic variant had a better survival rate over patients with G/G and T/T variants *P* = .38) (Figure 2A). For patients who were identified with T/G polymorphic variant after 1600 days survival rates amounted to 56.9%, and for those who were determined by T/T polymorphic variant survival rates reached 48.8%. After approximately 800 days of observation, survival rates of patients with different polymorphic variants were almost identical: T/T 53.5% and T/G 56.9%. *MDM2* gene polymorphic G/G variant was identified in only 6 examined samples, therefore they were not included in this survival analysis.

Survival was 55.4% of patients with *MDM4*c.1q32 A>C gene SNP A/A and 48.5% with SNP A/C at 1600 days post-surgical operation, however, it showed no significant differences in survival groups regarding all tested polymorphic variants (*P* = .84) (Figure 2B). C/C polymorphic variant was identified in only 7 examined samples, therefore they were not included in this survival analysis. C/T and C/C polymorphic variants of the *MTHFR*c.677 C>T resulted in similar survival rates as the ones observed in the same test group after 1600 days (respectively 47.4% and 51.0%, *P* = .66) (Figure 2C). T/T polymorphic variants were identified only in 4 samples. As for *CASP8*c.-652 6N gene SNP ins/ins survival was 54.2% and SNP ins/del survival was 54.4% at 1600 days post-surgical operation (*P* = .99) (Figure 2D). Del/del allelic variant was identified only in 5 LC samples, therefore the statistical analysis was not performed. Survival differences between *CCR5*-Δ32 gene SNP groups were not significant, as SNP wt/Δ32 survival was 61.9% and SNP wt/wt survival was 51.6% at 1600 days post–surgical operation (*P* = .59) (Figure 2E). Δ32/Δ32 polymorphic variant was found in only 2 samples, so statistical analysis was not performed.

### Survival analysis according to *MDM2*c.-5+309 G>T*, MDM4*c.1q32 A>C*, MTHFR*c.677 C>T*, CASP8*c.-652 6N ins/del*, CCR5*- Δ32 genes SNPs in patients with early and late stages of cancer

Patients who were diagnosed with stage I, II, III, and IV LC had significantly different survival curves (*P* < .01), and survival rates on 1494 day of study were 72.2%, 37.4%, 39.2%, 20.0%, respectively. Overall, analysis showed that patient survival rates had a trustworthy correlation with the staging of LC. After statistical analysis of tested genes SNPs, no reliable differences were found between patients diagnosed with early (I-II) and late (III-IV) stages of LC (Figure 2). However, after 1418 days survival rates of patients diagnosed with *MTHFR*c.677 C>T gene SNP C/C and stage I-II LC was 62.7%, while 42.0% for C/T patients (*P* = .17) (Figure 2C). Patients with stage III-IV LC who were identified with gene *MDM2*c.-5 + 309 G>T SNPs survival differences were not statistically significant, T/T patients survived post operation up to 800 days, while 43.9% of T/G patients survived until the end of the observation (*P* = .23) (Figure 2A). At the end of the study, survival rates were more than 10% different among the patients diagnosed with stage I-II and identified with SNP of CASP8c.-652 6N gene: 70.6% in case of ins/ins and 53.0% in ins/del (*P* = .33) (Figure 2D). Survival rates of patients diagnosed with III-IV stage of LC and SNPs of *CCR5*-Δ32 gene were 26.5% in case of wt/wt and 46.3% in wt/Δ32 (*P* = .33) (Figure 2E).

### Cluster analysis of *TP53, MDM2, MDM4, MTHFR, CASP8, CCR5* genes polymorphic variants

**Figure 3:**
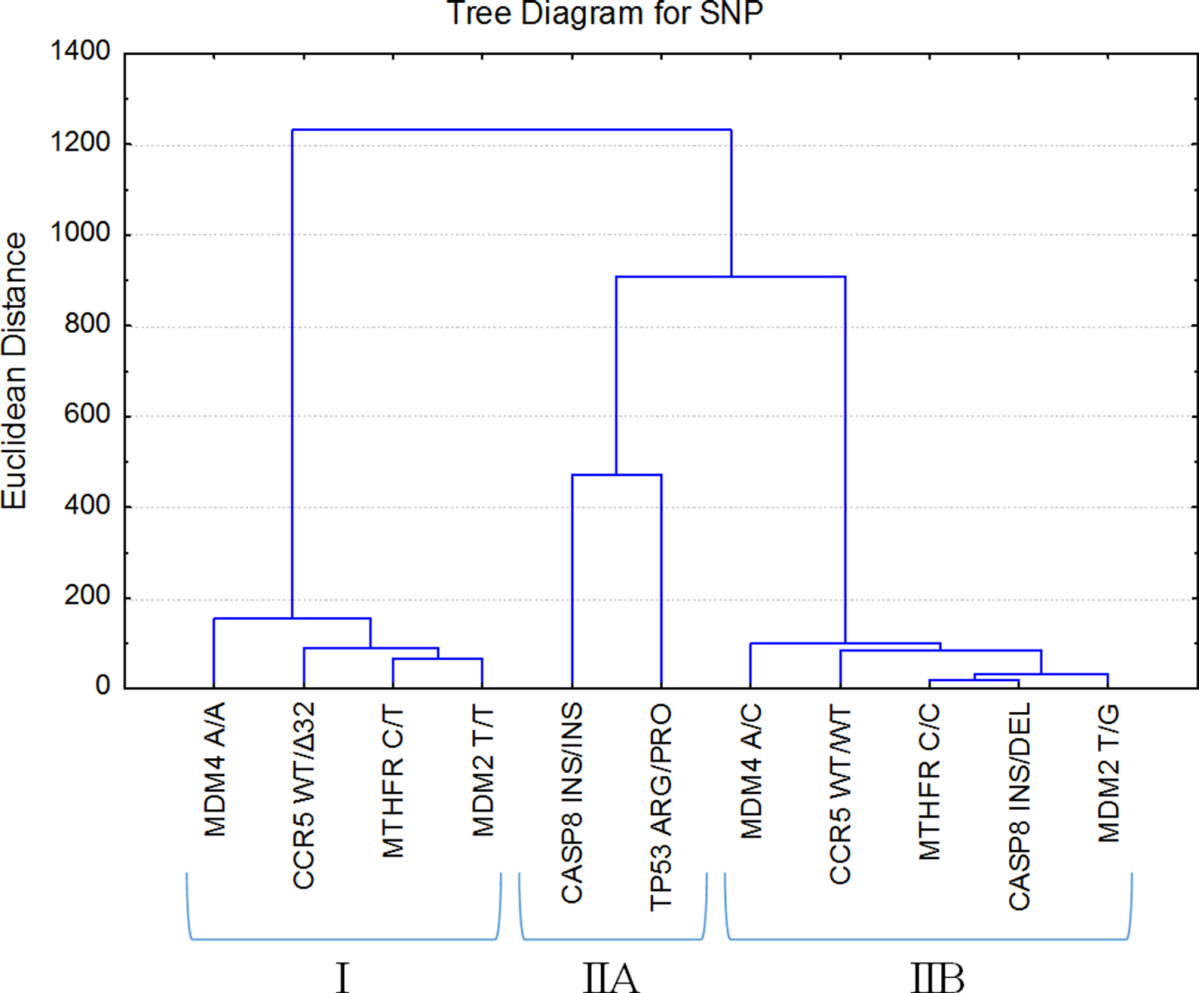
SNP clustering on the average survival median (q_1_-q_3_) and mortality rates Euclidian distances and Ward’s method were used for groups merging. SNP average survival median chart was formed on patients who were identified with *MDM4* A/A, *CCR5* wt/Δ32, *MTHFR* C/T, *MDM2* T/T, *CASP8* ins/ins, *TP53* Arg/Pro, *MDM4* A/C, *CCR5* wt/wt, *MTHFR* C/C, *CASP8* ins/del, and *MDM2* T/G polymorphic variants.

Finally, cluster analysis was performed on the basis of the patients’ mortality rates and survival times to determine which of the analyzed SNPs resulted in the best patient survival. Table 2 and Figure 3 provide the results of cluster analysis. The highest mortality rate had groups of patients who were identified as *MDM2* T/T and *CCR5* wt/wt (43.4%), *MDM4* A/C and *MTHFR* C/C (43.3%) polymorphic variants. The lowest mortality rates were observed in patients who were identified as *CCR5* wt/Δ32, *MDM2* T/G, *MTHFR* C/T, and *CASP8* ins/del genetic polymorphic variants with numerical expressions 30.7%, 38.4%, 39.4%, and 40.0%, respectively. For patients who were identified as *MDM4* A/A, *CASP8* ins/ins, *TP53* Arg/Pro polymorphic variants, mortality rates were 40.7%, 42.3%, and 42.5%, respectively.

**Table 2:** SNP-dependent survival rate (%) in group of patients who did not survive until the end of the study

Cluster	I				IIA		IIB				
SNP	*MDM4* A/A	*CCR5* wt/∆32	*MTHFR* C/T	*MDM2* T/T	*CASP8* ins/ins	*TP53* Arg/Pro	*MDM4* A/C	*CCR5* wt/wt	*MTHFR* C/C	*CASP8* ins/del	*MDM2* T/G
%^*^	40.7	30.7	39.4	43.4	42.3	42.5	43.3	43.4	43.3	40.0	38.4
M^**^ **(IQR)**	355 (353)	413 (492)	375 (518)	437 (516)	723 (716)	711 (153)	572 (416)	498 (437)	557 (423)	542 (412)	572 (398)

^*^Mortality rate

^**^Survival median (IQR (Q_3-_Q_1_)).

It should be emphasized that clasterisation of each SNP mostly depended on survival time median, *Q*1 and *Q*3 (represented in Table 2 as interquartile range (IQR)). Cluster analysis identified two patient groups that contained distinct variants of gene polymorphism. In the first group (I) patient survival was up to 437 days and in the second group (II) survival of patients was beyond 437 days. Cluster covering group of patients with better survival rate (II) reveals two more groups: the patients with *CASP8* ins/ins, *TP53* Arg/Pro gene polymorphism variants (IIA) and with two times longer survival than patients from cluster (I); and the second group (IIB) includes patients with identified *MDM4* A/C, *CCR5* wt/wt, *MTHFR* C/C, *CASP8* ins/del, *MDM2* T/G gene polymorphism variants and their survival was between 498–572 days.

**Figure 4:**
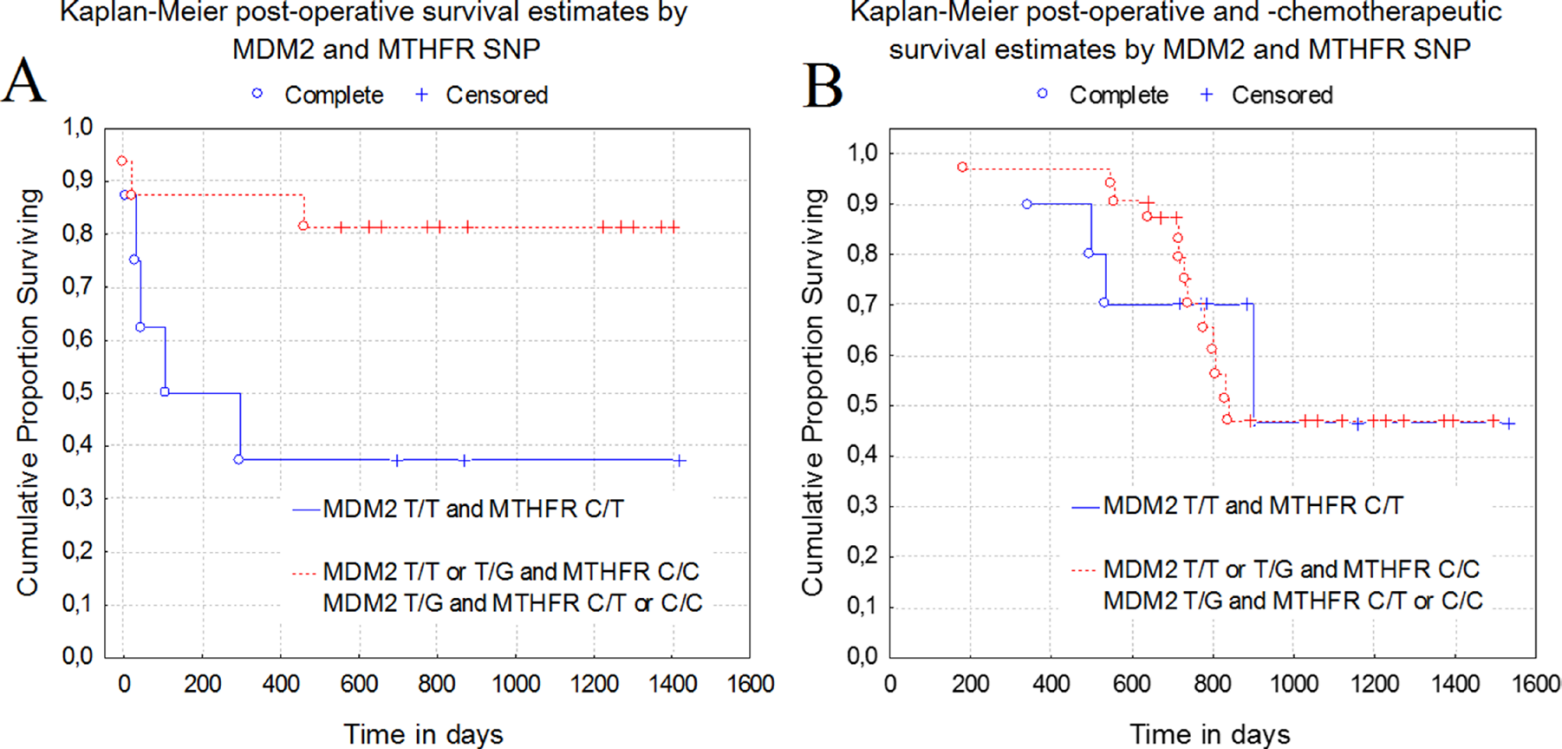
Relation between the survival and the combination of *MTHFR*, *MDM2* genes polymorphic variants (**A)** - Combination of *MTHFR* C/T and *MDM2* T/T was significantly related to the poor patient survival after lung cancer surgery (*P* = .04). (**B**) - No significant link (*P* = .96) was detected between the combination of *MDM2* T/T, *MTHFR* C/T and combinations of other polymorphic variants of *MDM2* and *MTHFR* genes in regard to survival of LC patients after chemotherapy or other therapeutic treatment.

Our cluster analysis showed that the genes in cluster I might be associated with the lowest survival rates. A more detailed analysis has shown that C/T polymorphism of the *MTHFR* gene and T/T polymorphic variant of *MDM2* gene are the most closely correlated with the poor survival of patients. The rate of both genes *MTHFR* C/T (χ2 = 2.4, df = 1, *P* = .12) and *MDM2* T/T (χ2 = 2.6, df = 1, *P* = .11) had a tendency to decrease in samples of patients diagnosed with stage III-IV cancer. This could be attributed to the worse survival of patients in the early stages of the disease. Therefore, it is worthwhile to check an importance of these genes in the first stages of the disease and in the period after the surgical treatment. Our results showed that the combination of polymorphic variants C/T of the *MTHFR* gene and T/T of the *MDM2* gene are significantly linked to the poor survival of LC patients after surgery (*P* = .04). Meanwhile, individual SNPs of genes have no significant effect on the survival of patients after surgery: *MTHFR* C/T (*P* = .38), *MDM2* T/T (*P* = .49). Moreover, no significant link (*P* = .96) was detected between the combination of *MDM2* T/T, *MTHFR* C/T and combinations of other polymorphic variants of *MDM2* and *MTHFR* genes in regard to survival of LC patients after chemotherapy or other treatments strategies applied. Patients with *MDM2* T/T and *MTHFR* C/T and who underwent surgery had 37.5% survival rate for one year, while it was 90.0% for patients with the same combination of SNPs and who received chemotherapy. Significance of the tested genes SNPs for the survival of lung cancer patients analysed with Cox model has not showed the single SNP effect, but SNPs combination of genes *MDM2* T/T, *MTHFR* C/T (*P* = .14) along with the stage of patients with lung cancer (*P* = .02) were associated with different periods of survival of lung cancer patients (Figure 4).

### HPV phylogenetic line identification

After PCR analysis HPV infection was identified only in 3 tumor samples of all 92 LC patients (3.3%). In one case HPV of A9 phylogenetic line (showing infection of 16, 31, 33, 35, 52, 58 types of HPV) was identified, in the other A5/A6 (51 and 56 types of HPV) was detected. In the third sample infection with two A9 and A7 (18, 39, 45, 59 types of HPV) phylogenetic lines were stated. However, the exact type of virus was not specified. But in all three samples the viral copy number in the cell was determined. In sample with A9 phylogenetic line the number of virus copies was 0.98 lg HPV copy/cell, in sample with A5/A6 phylogenetic lines the viral load comprised 2.2 lg HPV copy/cell. And in sample with two A9 and A7 phylogenetic lines number of virus copies amounted to 2.7 HPV copy/cell. It is interesting to note that two out of three patients who were identified as HPV infected were smokers. One smoker was infected with A9, another with both A9 and A7 phylogenetic line viruses. Men patients harbored A9 HPV, whereas women A9, A7, A5/A6 HPV phylogenetic lines. Moreover, in all three HPV positive samples TP*53*c.215 G>C Arg/Pro, *CASP8*c.-652 6N ins/del, and *CCR5*-Δ32 wt/wt polymorphic variants were detected. In the sample with A7 and A9 phylogenetic line viruses *MDM4* A/A and *MTHFR* C/C SNPs were identified. Others SNPs are shown in Table 3.

**Table 3:** Analysis of *TP53*, *MDM2*, *MDM4*, *MTHFR*, *CASP8*, *CCR5*-*Δ32* genes polymorphic variants of HPV positive lung cancer patients

No.^*^	Phylogenetic line	Viral copies^**^	*TP53c*.215 G>C (Arg72Pro)			*MDM2c*.5+309 G>T			*MDM4c*.1q32 A>C			*MTHFRc*.677 C>T			*CASP8c*.-652 6N ins/del			*CCR5c*.-∆32		
			Arg/Arg	Arg/Pro	Pro/Pro	T/T	T/G	G/G	A/A	A/C	C/C	C/C	C/T	T/T	Ins/ins	Ins/del	Del/del	Wt/wt	Wt/∆32	∆32/∆32
1	A9	0.98																		
2	A9,A7	2.7																		
3	A5/6	2.2																		

^*^Patient identification number

^**^Number of copies of the virus expressed as lg HPV copy/cell.

## DISCUSSION

Literature research data on relationship between SNPs of genes *TP53*c.215 G>C (Arg72Pro), *MDM2*c.-5 + 309 G>T, *MDM4*c.1q32 A>C, *MTHFR*c.677 C>T-652, *CASP8*c.6N ins/del, *CCR5*-Δ32 and patients with LC survival rates were highly controversial. All our samples of LC patients were investigated to identify *TP53*c.215 G>C (Arg72Pro), *MDM2*c.-5+309 G>T, *MDM4*c.1q32 A>C, *MTHFR*c.677 C>T-652, *CASP8*c. 6N ins/del, and *CCR5*-Δ32 genes polymorphic variants.

Our study showed that heterozygous Arg/Pro polymorphic variant of gene *TP53*c.215 G>C (Arg72Pro) dominates and the frequency distribution is as follows: Arg/Pro 95.65%, Arg/Arg 4.35%. The high difference in the distribution of these alleles was shown in other populations: in the Iranian population, gene *TP53* Arg/Arg, Arg/Pro, Pro/Pro frequencies were respectively 42.6%, 49.6%, and 7.8% [42], while in the Korean population respectively 37.0%, 46.2%, and 16.7% [43]. *Sreeja et al.* Kaplan-Meier survival analysis showed a significant difference in LC patients’ survival between *TP53* variant genotypes and overall survival. Cox regression analysis showed *p53* Arg72Pro heterozygous genotype was overall an independent prognostic factor, suggesting Pro72Pro genotype to be a potential risk factor favoring the development of lung carcinoma [44].

Published data on the association between *MDM2* 309 T/G polymorphism and risk for LC development are inconclusive [45]. *Javid et al.* revealed that *MDM2* gene G/G polymorphic variant might be associated with increased expression of *MDM2*, which operates p53 expression changes [46]. *Chua et al.* published a study of Singaporean non-smokers LC patients. It was revealed that in female samples not the G/G, but T/T polymorphism in position 309 of gene *MDM2* increases the risk 2.1 times, and combination with the *TP53* Pro/Pro allele increases the risk 2.5 times. There was, however, no effect of either polymorphism on age at diagnosis of LC or on overall survival [47]. *Enokida et al.* published a case-control study showing gene *MDM2* T/T, T/G, G/G polymorphic variants frequencies in samples to be 21.1%, 49.7%, and 30.2%, respectively [48]. Our study polymorphisms frequencies of *MDM2*c.-5+309 G>T gene T/T, T/G, G/G showed that T/T polymorphic variant dominates and frequencies are 50.00%, 43.48%, 6.52%, respectively. *Dong et al.* found that the *MDM2* SNP309 (rs2279744) GT/TT genotypes are associated with a significantly worse survival [49]. *Enokida et al.* found that the overall survival of adenocarcinoma patients with pathological stage I disease and the *MDM2* T/T genotype was significantly shorter than that of those with the T/G or G/G genotypes (*P* = .02) [50]. Our study showed no difference in mortality rate of patients harbouring gene *MDM2* T/G or T/T polymorphic variant (respectively 38.4% and 43.4%). The similar tendency is observed in the assessment of survival time (respectively 572 and 437 days). However, patients with advanced stages of LC and T/T variant of the *MDM2* gene has the worst overall survival rate in the end of follow-up, but data were not statistically significant (Figure 2).

Analyses suggested that the rs4245739 polymorphism was significantly associated with overall cancer risk [51]. In *Yang et al.* study rs4245739 A/C genotype showed increased overall survival of LC patients in comparison to those with A/A genotype (*P* = .04) [52]. Our research results indicate that A/A polymorphic variant of gene *MDM4*c.1q32A>C dominates in LC samples. The frequency of A/A, A/C, C/C distribution is respectively 59.8%, 32.6%, and 7.6%. We showed that for patients identified with gene *MDM4* A/A SNP mortality rate is lower than with A/C (A/A 40.7%, A/C 43.3%). Patients with A/A polymorphic variant have 1.6 times shorter survival rate after surgery compared with patients who were identified with A/C polymorphic form (355 days for A/A and 572 days for A/C). However, the data also were not statistically significant.

Definite conclusions cannot be drawn from studies analysing the relation between *MTHFR* C677T polymorphism and LC risk [53, 54, 28, 55]. *Cui et al.* determined frequencies of *MTHFR*c.677 polymorphic C/C, C/T, T/T variants to be 34.5%, 48.5%, and 17.0%, respectively. *MTHFR* 677 C/T and T/T genotypes showed a weak protective effect on LC development, compared with homozygous C/C genotype, although these results were not statistically significant [56]. *Cheng et al.* showed *MTHFR*c.677 C>T polymorphic variants C/C, C/T, T/T distribution to be respectively 27.7%, 35.1%, 37.2% and found T/T genotype link with LC [69]. *Al-Motassem et al.* established distribution of *MTHFR*c.677 C>T polymorphic variants as follows: C/C 59.6%, C/T 33.0%, and T/T 7.4%. Authors announced that patients who have C/C polymorphic variant also have an increased risk for LC [57]. Similar results on *MTHFR*c.677 C>T SNP distribution in LC patients were showed in our study: C/C polymorphic variant was dominating in our samples, C/C, C/T, T/T polymorphisms frequencies were respectively 58.7%, 37.0%, and 4.4%. However, for our patients who were identified with *MTHFR* C/T gene polymorphic variant mortality rate is almost the same as for those with C/C polymorphic variant (39.4% and 43.3%, respectively), but their survival time stands out (respectively 375 and 557 days). Moreover, patients with advanced cancer stages (III-IV) and harbouring C/T polymorphism of the *MTHFR* gene had the shortest survival rate in regard to other *MTHFR* SNP variants (χ2 = 2.4, *P* = .12, df = 1). This difference was not statistically significant as well.

In the analysed literature, SNPs studies of *CASP8* -652 6N gene have shown no clear association with LC. *Sun et al.* showed that *CASP8* -652 6N del polymorphism is associated with decreased LC development risk in China’s population [58]. *Ji et al.* found that patients with *CASP8* -652 6N del polymorphism have greater susceptibility for LC development [32]. In our study, *CASP8*c. 6N-652 ins/del polymorphic ins/ins, ins/del, del/del polymorphisms frequency analysis showed that ins/del dominates and frequencies are respectively 28.3%, 66.3%, and 5.4%. We showed that mortality rate is lower for patients identified with *CASP8* ins/del polymorphic form (40.0%) than for patients with ins/ins polymorphic variant (42.3%), but they survived shorter (542 days) than patients who were identified as ins/ins polymorphic variants (723 days).

To our best knowledge, there are no literature data on *CCR5-*Δ32 polymorphic variants interface with LC development. It is known that patients identified with *CCR5*-Δ32/Δ32 genotype have a 4.58 times higher risk for HPV infection [35]. Due to these reasons, we analysed possible combined effect of HPV infection and *CCR5-*Δ32 in LC cancerogenesis. Our results indicated that wt/wt polymorphic variant of gene *CCR5-*Δ32 dominates in samples and wt/wt, wt/∆32, ∆32/∆32 frequency distribute respectively 83.7%, 14.1%, and 2.2%. We showed that for patients identified with gene *CCR5* wt/Δ32 polymorphic variant mortality rate is lower (30.7%) than for wt/wt polymorphic form (43.4%), and their survival is lower (413 days) than for patients identified with wt/wt polymorphic form (498 days). HPV infection, as mentioned above, was found only in 3 analysed samples, so statistical analysis of combined effect on HPV and analysed genes SNPs was not performed.

Other authors published several studies on combined effect of different SNPs and effect on LC development and disease progression. One of the study showed that combined *TP53* Pro/Pro and *MDM2* G/G genotypes had a supermultiplicative interaction with respect to lung adenocarcinoma risk [59]. P53 pathway offers multiple molecular targets for screening small molecules as potential cancer therapies. Within the past decade number of new small molecules has been identified to target *p53, MDM2* or *MDM4* polymorphic variants, some of which have been further advanced to the stage of clinical trials [60]. SNPs investigated in our study could be used as molecular targets in drug screening for potential novel LC therapies. Overall, detection of different polymorphic variants could help the physicians to evaluate more precisely the prognosis of LC patients or to apply more specific therapy, however, many authors conclude that these observations require further investigations with larger groups in order to identify potential markers for LC better prognosis and targeted therapy.

Combined effect of *MTHFR* and other genes (*TS* and *ERCC1*) polymorphisms on effectiveness in first-line platinum and pemetrexed therapy in NSCLC patients was evaluated in Poland [70, 71]. Authors showed that early LC progression was observed significantly more frequent in patients with T/T genotype than in patients with C/C or C/T genotypes of *MTHFR* 677C>T polymorphism, however, progression-free and overall survival were not affected by *MTHFR* gene polymorphism. After combined analysis of *TS* VNTR (variable number of tandem repeats)and *MTHFR* 677C>T polymorphisms, a shorter progression-free survival in patients harboring C/C in *MTHFR* gene was shown [70]. Later authors concluded that carriers of 3R in *TS* gene and C/C genotype of *MTHFR* gene have significantly shorter overall survival (HR = 3.07; *P* =.003) and significantly higher risk of death (HR = 3.85; *P* < .005) [71]. Earlier *Smit et. al* [72] showed, that LC patients with T/T polymorphic variant of *MTHFR* gene have longer progression-free survival time compared to patients with C/C or C/T polymorphic variants. *Li et*. al [73] showed that *MTHFR* heterozygotes yield better clinical benefit (*P* =.03) after platinum based chemotherapy, however, overall survival and progression-free survival of LC patients do not differ regarding *MTHFR* polymorphic variants. In our study we performed cluster analysis which showed that the combination of *MTHFR* C/T and *MDM2* T/T are significantly linked to the poor survival of LC patients after surgery (*P* = .04). However, the effect of SNPs of our investigated genes on the effectiveness of chemotherapy was not evaluated in our study. Nevertheless, genetic factors (alone or in combination) may have a high predictive and prognostic value for LC survival after surgery or chemotherapy applied.

Regarding viral infection and lung cancer, the first evidence that there is a link between HPV infection and bronchial lesions was determined in 1979 by Rubel and Reynolds [2]. *Syrjänen et al.* in 1979 introduced the idea that HPV infection may be one of the factors involved in process of lung carcinogenesis [41]. Over the past years, several studies analysing HPV infection and LC were carried out. HPV infection is established from 0% to 36% in patients of Western world with lung adenocarcinoma and from 9% to 78% in Asia population [38]. Recently, *Sarchianaki et al.* survey data showed that HPV types identified in LC patients could be such as 16, 11, 33, 31, 18, 6, 59. The most commonly found type is 16 (42.1%) and 11 (15.8%) [61]. In our study, 2 out of 3 patients with HPV identified in LC samples were females, one infected with A9, A7, A5/A6 phylogenetic line HPV, and the other one harboured viruses belonging to two phylogenetic groups. Based on these findings we suppose that HPV could spread from cervical epithelium through blood or lymph. However, we did not test cervical HPV infection and exact type of the virus for these women. In all three HPV-positive samples *TP53*c.215 G>C Arg/Pro, *CASP8*c.-652 6N ins/del, *CCR5-*Δ32 wt/wt polymorphic variants occurred. *Jain et al.* study of India’s population showed that out of 40 LC patients 2 were infected with HPV and were identified with *TP53*c.215 G>C (Arg72Pro) gene Arg/Arg polymorphic variant (apart from patients with confirmed lung IV cancer stage, Arg/Pro polymorphic variant dominated in these samples) [62]. Lately, *Amaral et al.* connection analysis of cervical lesions, HPV, oral contraceptive, and *MDM2*c.-5+309 G>T polymorphism found that there is a link between high-grade cervical lesions, contraceptive use, and *MDM2* gene T/G polymorphic variant [63]. To our knowledge, we are the first to analyze the interactions between *MDM2*c.-5+309 G>T, *MDM4*c.1q32 A>C, *MTHFR*c.677 C>T-652, *CASP8*c. 6N ins/del, *CCR5*-Δ32 polymorphic variants, lung cancer, and HPV. Unfortunately, due to the small number of HPV infected patients in our study it was not possible to evaluate combined effect of HPV infection and different genes polymorphisms on the survival of LC patients. Therefore, here we conducted a study of *TP53*c.215 G>C (Arg72Pro), *MDM2*c.-5+309 G>T, *MDM4*c.1q32 A>C, *MTHFR*c.677 C>T-652, *CASP8*c.-652 6N ins/del, and *CCR5*-Δ32 polymorphic variants frequency analysis in LC samples (*n* = 92), regardless of the HPV infection. It is worthwhile to continue research in this direction with larger patient groups in order to clarify HPV implication in LC.

Finally, there is a link between specific SNPs and LC patient survival frequency and time, meanwhile the combination of specific SNPs showed a statistically significant measure. In conclusion, statistically significant (*P* = .04) link was determined between the poor survival of LC patients after surgery with combination of polymorphic variants C/T of the *MTHFR* and T/T of the *MDM2* genes, whereas individually these SNPs do not show significant relationship with our LC patients survival. However, it is important to notice that genetic factors (alone or in combination) may have a high predictive and prognostic value for LC survival after different treatments strategies applied.

## MATERIALS AND METHODS

### Study subjects and design

In the period from September of 2012 to December of 2014 92 patients (76 male and 16 female) with diagnosis of primary LC from Department of Thoracic Surgery at the National Cancer Institute (Vilnius, Lithuania) were asked to participate in the study. Study protocol was accepted by the Vilnius Regional Committee for the Biomedical Research (11-06-2013 No. 158200-13-638-204). All patients signed Informed consent before the surgery. Patients were included into the study according to the following criteria: all of them had primary lung cancer and none of them had received radiotherapy or chemotherapy prior to surgery. After surgery all tumors were examined by the pathologist and the diagnosis was confirmed by expert pathologists. Remaining material after the diagnosis was collected to the Biobank at the National Cancer Institute and stored till the scientific examination. All samples previously had been analyzed for the presence of HPV. Then the SPN analysis of various previously described genes was performed.

### DNA extraction

DNA was purified by organic extraction method according to the approved standard operating procedures at the Biobank of the National Cancer Institute. Firstly, after pathology diagnosis, all tumor tissue samples were cut into small pieces (30–50 mg) and were snap froze in the liquid nitrogen. Control cut was also performed for the evaluation of amount of cancer cells in the sample. Before DNA extraction tumor sample was crushed and melted in liquid nitrogen. Melted material was placed in a test-tube, lysis buffer (50 mM Tris HCl, pH 8.0; 200 mM NaCl; 20 mM EDTA, pH 8.0, 1% SDS) and proteinase K solution (10 mg/ml) were added. Tube was incubated in 55ºC for 12 hours. DNA purification was carried out with phenol-chloroform solutions and precipitation with 70% and 96% ethanol solutions. After that DNA was dried. Later the dried DNA was dissolved in 500 µl of sterile distilled water at 4°C. DNA purification quality was measured using spectrophotometer (NanoDrop™ 2000, Thermo Scientific). The quantitative DNA analysis was performed by measuring optical density at 280 nm wavelength. According to the ratio of A260/A280, isolated DNA of all samples used in this study was of adequate quality being in a range from 1.7 to 1.9.

### Genotype analysis

From each tumor sample extracted DNA was used for PCR analysis of *TP53* Arg72Pro (rs1042522), *CASP8*-652 6N (rs3834129), and *CCR5* ∆32 (rs333) genes. For gene *TP53* Arg72Pro (rs1042522) allele 4 different specific primers were used: Arg allele forward: 5′→ TCCCCCTTGCCGTCCCAA→3′ and reverse: 5′→CTGGTGCAGGGGCCACGC→3′, Pro allele forward: 5′→GCCAGAGGCTGCTCCCCCC→3′ and reverse: 5′→ CGTGCAAGTCACAGACTT→3′, which had revealed the following results of 141 bp and 177 bp products [64]. Likewise, for *CASP8*-652 6N (rs3834129) gene 2 primers were used: forward: 5′→AGTGAAAACTTCT CCCATGGCCTC→3′ and reverse: 5′→ GATTGATACTG GCACAGTATACTTACC→3′, and two specific primers: (Insertion allele): 5′→GTAATTCTTGCT CTGCCAAGCTG→3′; (Deletion allele) 5′→CCAAGGTCA CGCAGCTAGTAAG→3′, which had revealed the following results of 139 bp, 291 bp, and 396 bp fragments [65]. And for *CCR5*- Δ32 (rs333) gene 2 specific primers were used: forward: 5′→ACCTGCAGCTCTCATTTTCC→3′ and reverse: 5′→GCAGATGACCATGACAAGCA→3′, which had revealed the results of 111 bp and 79 bp fragments [35]. After PCR reactions with specific primers, for product visualisation LabChip GX/GX II Touch (Perkin Elmer) capillary electrophoresis method was used to identify all studied genes polymorphic variants.

**Figure 5:**
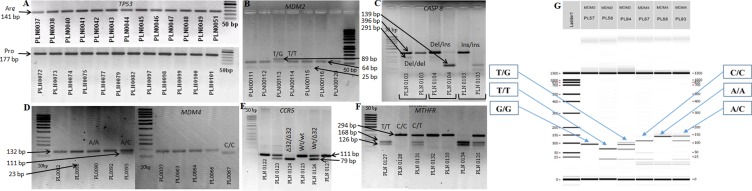
*TP53, MDM2*, *MDM4*, *MTHFR*, *CASP8*, and *CCR5* genes polymorphic variants of lung tumor tissue using agarose electrophoresis and LabChip GX/GX II Touch capillary electrophoresis gel (**A**) - *TP53* polymorphic variants (141bp - Arg; 177bp - Pro). (**B**) - SNPs of gene *MDM2* (89bp – G/G; 64bp, 25bp – T/T; 89bp, 64bp, 25bp – T/G). (**C)** - SNPs of gene *CASP8* (396bp, 291bp – del/del; 396bp, 291bp, 139bp – del/ins; 396bp, 139bp – ins/ins). (**D)** - SNPs of gene *MDM4* (132bp – A/A, 132bp, 111bp – A/C, 111bp – C/C). (**E**) – SNPs of gene *CCR5* (79bp - ∆32/∆32; 111bp – wt/wt; 111bp, 79bp – wt/∆32). (**F)** – SNPs of gene *MTHFR* (168pb, 126bp – T/T; 294bp – C/C; 294bp, 168bp, 126bp – C/T). (**G)** - *MDM4* and *MDM2* gene polymorphic variants of lung tumor tissue using LabChip GX/GX II Touch capillary electrophoresis method. On the left side *MDM2*c.-5 + 309G> T variants are showed: in PL57 sample G/G 89 bp was found; in PL58 sample two fragments of 64 bp and 25 bp were found, reflecting the presence of the T/T genotype; and all three bands (89, 64, 25 bp) showed the presence of the T/G genotype in PL94 sample. On the right side *MDM4*c.1q32 A> C polymorphic variants are showed: A/A 132 bp (PL88 sample); C/C 111 bp, 23 bp (PL67 sample); A/C 132 bp, 111 bp, 23 bp (PL93 sample).

*MDM2* T309G (rs2279744), *MDM4* (rs4245739), and *MTHFR* (rs1801133) genotypes were determined by polymerase chain reaction – restriction fragment length polymorphism (PCR-RFLP). A 89 bp of fragment covering *MDM2* gene was amplified by a set of primers: forward: 5′→TTCGGAGGTCTCCGCGGGAGTTCAG→3′ and reverse: 5′→TGCGATCATCCGGACCTCCCGCGTC→3′ [66]. The PCR product was digested with restriction enzyme TaqI (FastDigest Enzyme, Thermo Scientific) at 65°C for 8 min. T/G genotype was identified by existence of 89 bp, 64 bp, and 25 bp fragments. The PCR product of gene *MDM4* (forward primer: 5′→AAGACTAAAGAAGGCTGGGG→3′, reverse primer: 5→TTCAAATAATGTGGCAAGTGACC→3′) (rs4245739) was digested with restriction enzyme MspI. Polymorphic variants were identified by existence of 134 bp, 111 bp, and 23 bp fragments [67]. Finally, the PCR product of gene *MTHFR* (rs1801133) (forward primer: 5′→CCTTGAACAGGTGGAGGCCAG→3′, reverse primer: 5′→GCGGTGAGAGTGGGGTGGAG→3′) [68] was digested with restriction enzyme Hinfl and the results of this study were fragments of 294 bp, 168 bp, 126 bp. All fragments were analysed by LabChip GX I Touch capillary electrophoresis and electrophoresis in agarose gel method. Example of fragments analysis is presented in the Figure 5.

### HPV A9, A7, and A5/A6 phylogenetic group detection

Phylogenetic group of HPV, viral copy number, and the total number of copies of the virus in cells were determined by RT-PCR. AmpliSens HPV screen-titre-FRT PCR kit (AmpliSens) was used to determine HPV A9, A7, and A5/A6 phylogenetic groups. The kit was composed of PCR-mix-1-FRT HPV A9, A7, and A5/A6 phylogenetic groups according to HPV *E1 - E2* gene-based primers. In A9 phylogenetic line HPV types 16, 31, 33, 35, 52, 58; in A7 phylogenetic line HPV types 18, 39, 45, 59; and in phylogenetic line A5/A6 HPV types 51 and 56 were attributed. Amplification was performed using the Rotor-Gene *Q* amplificator and Rotor-Gene *Q* software (version 2.1.9.9). Endogenic control with β–globin was performed for each PCR cycle. Number of copies of the virus in the cell was calculated using the formula: log (HPV DNA copies/human DNA copies) × 200,000 = log (HPV of 100,000 cells).

### Statistical analysis

Statistical comparisons among groups were performed by Chi-square test. Survival was estimated by Kaplan-Meier method. The statistical difference between the survival curves was determined using the log-rank test. The variables with log-rank test *P* value < 0.2 were selected for Cox regression analysis. For SNPs analysis clusterisation in a multi-dimensional space (mortality rate; quartile 1 (Q1), median (M), and quartile 3 (Q3) of patient survival time) was performed computing Euclidean distances between objects and linking several objects by Ward’s method. A *P* value of < .05 was considered statistically significant. Statistical analysis was performed using SigmaPlot 12.3 and STATISTICA 10.0 software.
